# An experimental validation of genomic selection in octoploid strawberry

**DOI:** 10.1038/hortres.2016.70

**Published:** 2017-01-11

**Authors:** Salvador A Gezan, Luis F Osorio, Sujeet Verma, Vance M Whitaker

**Affiliations:** 1 School of Forest Resources and Conservation, University of Florida, 363 Newins-Ziegler Hall, PO Box 110410, Gainesville, FL 32611-0410, USA; 2 Gulf Coast Research and Education Center, University of Florida, 14625 CR 672, Wimauma, FL 33598, USA

## Abstract

The primary goal of genomic selection is to increase genetic gains for complex traits by predicting performance of individuals for which phenotypic data are not available. The objective of this study was to experimentally evaluate the potential of genomic selection in strawberry breeding and to define a strategy for its implementation. Four clonally replicated field trials, two in each of 2 years comprised of a total of 1628 individuals, were established in 2013–2014 and 2014–2015. Five complex yield and fruit quality traits with moderate to low heritability were assessed in each trial. High-density genotyping was performed with the Affymetrix Axiom IStraw90 single-nucleotide polymorphism array, and 17 479 polymorphic markers were chosen for analysis. Several methods were compared, including Genomic BLUP, Bayes B, Bayes C, Bayesian LASSO Regression, Bayesian Ridge Regression and Reproducing Kernel Hilbert Spaces. Cross-validation within training populations resulted in higher values than for true validations across trials. For true validations, Bayes B gave the highest predictive abilities on average and also the highest selection efficiencies, particularly for yield traits that were the lowest heritability traits. Selection efficiencies using Bayes B for parent selection ranged from 74% for average fruit weight to 34% for early marketable yield. A breeding strategy is proposed in which advanced selection trials are utilized as training populations and in which genomic selection can reduce the breeding cycle from 3 to 2 years for a subset of untested parents based on their predicted genomic breeding values.

## Introduction

Genomic selection (GS) was originally proposed in animal breeding^1^ as a methodology to efficiently use the vast amount of marker information that was generated by new DNA technologies to predict the genetic merit of individuals traditionally estimated by best linear unbiased prediction, BLUP.^2^ GS uses a training population of individuals with known phenotypes and marker data to build a model for the prediction of performance in a population of untested individuals based on marker data. GS uses this marker data in two different ways: by using markers to model relationships between individuals or by estimating the effects of each marker on the trait of interest, some of which are presumed to be in linkage disequilibrium (LD) with relevant quantitative trait loci (QTL). The predictor of an individual phenotype is the genomic estimated breeding value (GEBV) obtained as the sum of all corresponding marker effects of the individual. GEBVs are then used to rank and select genotypes, without phenotypic data, for the next generation of breeding.^3^

Genomic selection has been studied or applied in several agricultural crops, trees and animal breeding programs,^4–7^ due to the increase in genetic gains that it provides compared with conventional methods. In a broad context, increases in selection response via GS are related to: (i) prediction of GEBV of untested individuals that do not have phenotypic records; (ii) shortening the breeding cycle;^5,8^ and (iii) improving the precision of estimates of genetic effects.^4^ However, other potential benefits can be obtained with the incorporation of GS into the breeding strategy, such as: (i) increasing selection intensity by having larger populations for prediction; (ii) reducing testing effort by eliminating partially or completely the establishment of some field experiments; (iii) better planning of crosses by more effective control of inbreeding and relatedness;^9^ (iv) predicting hard-to-measure traits using correlated traits as predictors;^10^ and (v) controlled reduction of genetic diversity in the short term.^9^

A variety of statistical methods to predict GEBV have been implemented to deal with the problem of a large number of genetic markers and a limited amount of phenotypic data, including parametric, Bayesian and non-parametric methods, which differ in their assumptions about the underlying genetic model.^7,11,12^ Differences between methods are usually small in empirical studies;^5^ however, some differences have been reported, mostly associated with differing genetic architectures among traits.^7^

At present, only a small number of studies have reported either the study or the application of GS in horticultural crops, including grapevine,^13^ apple,^8,14^ sugar beet^15^ and tomato.^16^ None have reported GS in strawberry (*Fragaria*×*ananassa*). Owing to the challenges presented by the allo-octoploid genetic constitution of cultivated strawberry, there has been a lack of genome-wide, high-throughput genotyping capability until the recent development of the Affymetrix Axiom IStraw90 SNP array^17^ and the Axiom IStraw35 array (Verma *et al.,*^18^ in press). With these resources in place, it is now possible to envision marker-assisted approaches to breeding for complex traits in strawberry. While certain traits in strawberry, particularly disease resistance, are controlled by major genes located to a single subgenome,^19–21^ other traits are controlled by alleles across multiple subgenomes.^22^ In the University of Florida strawberry breeding program, which develops cultivars for annualized winter and early-spring production systems, the genetic architectures of several complex yield and fruit quality traits^23^ have been described, for which no significant QTL or only a few minor-effect QTLs have been detected.^18^

The objective of this research was to experimentally evaluate different GS approaches for octoploid strawberry and to define a future breeding strategy for its operational implementation, using the University of Florida strawberry breeding program as an example. The focus of this study was on the use of datasets from different trials and years to perform true validations, in which a model is trained in a given trial/year and applied in a test population from a separate trial/year. A secondary objective was to estimate linkage disequilibrium in the germplasm tested in order to inform interpretations of GS results and GS implementation.

## Materials and methods

### Plant material and field trials

The strawberry genotypes included in this study were a representative selection of parents, current named cultivars and advanced selections of the strawberry breeding program at the University of Florida, Institute of Food and Agricultural Sciences. Four clonally replicated field trials corresponding to stage 1 (T1 trials, consisting of unselected seedlings generated from a circular mating design), and stage 2 (T2 trials, consisting of advanced selections selected as seedlings in previous years) were established at the Gulf Coast Research and Education Center in Balm, Florida, USA, during the 2013–2014 (T1/2013, T2/2013) and 2014–2015 (T1/2014, T2/2014) seasons. There were 62 full-sib families arising from 22 parents in a circular mating design in trial T1/2013 and 60 full-sib families arising from 31 parents in a circular mating design in trial T1/2014. However, the total number of families tested in these trials was 76 and 70, respectively, as they included other selected crosses and control checks. The advanced selections from T2/2013 originated from 122 crosses among 86 parents, and those from T2/2014 originated from 109 crosses among 64 parents. Several advanced selections and control checks were common among pairs of trials, and there were many common ancestors among the genotypes evaluated (Table 1).

Germinated seedlings from the mating designs (T1 trials) were transported to a summer nursery near Monte Vista, Colorado, USA, for further clonal propagation by runners. Before establishment, all individual plants were weighed such that transplant weight (g) could be used as a covariate for a given trait. In contrast, runners and crowns from the advanced selections (T2 trials) were propagated by Crown Nursery near Macdoel, California, USA, for clonal propagation and transplants were not weighed. Thus, T1 and T2 trials differed in propagation source.

### Experimental design

For each trial, clonally propagated genotypes were arranged in a randomized complete block design. T1 trials from both seasons had three clonal replicates per individual and three raised beds per replicate block, with each bed subdivided into eight subplots, each with a common control to account for environmental variation along the bed. T2 trials had five clonal replicates per individual, one raised bed per replicate block and seven to nine subplots. In all trials genotypes were established as single-plant plots. The total number of individuals tested in the field for each trial is presented in Table 1. The test sites were prepared and maintained following standard commercial practices for west-central Florida, USA. Fertilization, weed management and pests and diseases control varied between seasons according to environmental conditions.

### Phenotyping and genotyping

Data for all traits was gathered on an individual plant basis. Fruit harvests were made weekly from the last week in November to the middle of March in both seasons. A total of six yield and fruit quality traits were evaluated in each trial. All ripe fruit were harvested and counted. Early marketable yield (EMY, g per plant) was obtained as the marketable weight of all fruit harvested until the end of January. Total marketable yield (TMY, g per plant) was calculated as the weight of all marketable fruit harvested until the middle of March. Average fruit weight (AWT, g per fruit) was estimated as the TMY divided by the number of marketable fruit. Total unmarketable (cull) fruit (TC, %) was evaluated as the proportion of total fruit that were unmarketable due to small size (<10 g), disease, malformation and any other quality defect. Soluble solids content (SSC, %) was calculated as the average of five measurements in each season. One ripe fruit per plant was hand-squeezed onto the prism of a handheld digital refractometer. Not all plants had ripe fruit at all harvest dates due to different ripening patterns.

A reference population of 1628 individuals, out of a total of 1834 genotypes, belonging to the four tests were genotyped with the Axiom IStraw90 SNP array. A total of 28 828 high-quality markers were segregating in these individuals. Quality control was implemented in which those markers with minor allele frequencies (MAF) <5% and with missing marker data >5% were eliminated. Hence, a total of 17 479 markers were available for further statistical/genetic analysis. Missing values for each of the markers (0.38%) were imputed based on the average allele frequency.

### Preliminary estimation of genetic parameters

The following generic linear model was fitted to each of the traits and trials separately using the raw data:

(1)y=1µ+wβ+Xr+Z1b(r)+Z2p(br)+Z3a+Z4f+Z5c(f)+e

where, *μ* is the overall mean; *w* is a covariate of the initial weight of a transplant, and *β* is its associated coefficient; **r** is a fixed replication effect; **b**(**r**) is the random effect of bed within replicate, with $b(r)˜MVN\left(0,\sigma_{b}^{2}Ι\right);$ **p**(**br**) is the random effect of plot within bed, with $p(br)˜MVN(0,\sigma_{p}^{2}I)$; **a** is the random additive effect of genotype, where $a˜MVN(0,\sigma_{a}^{2}A)$; **f** is the family random effect, with $f˜MVN(0,\sigma_{f}^{2}I)$; **c**(**f**) is the random effect of clone within family, with $c(f)˜MVN(0,\sigma_{c}^{2}I)$; and **e** is the residual term, with $e˜MVN(0,\sigma_{e}^{2}I)$. The matrix **A**, of dimension 3951×3951, is the numerator relationship calculated from pedigree information consisting of all tested individuals, parents and their ancestors, **1** is a vector of ones, **X** and **Z** are incidence matrices, and **I** is an identity matrix.

Narrow-sense heritability (*h*^2^) and broad-sense heritability (*H*^2^) for the above raw data analyses were calculated for the above fitted model using the expressions: $h^{2}=\sigma_{a}^{2}/\sigma_{t}^{2}$ and $H^{2}=\sigma_{g}^{2}/\sigma_{t}^{2}$, where $\sigma_{t}^{2}=\sigma_{b}^{2}+\sigma_{p}^{2}+\sigma_{a}^{2}+\sigma_{f}^{2}+\sigma_{c}^{2}+\sigma_{e}^{2}$ and $\sigma_{g}^{2}=\sigma_{a}^{2}+\sigma_{f}^{2}+\sigma_{c}^{2}$. In addition, an *ad-hoc* narrow $\left(h_{\bar{c}}^{2}\right)$ was obtained as $h_{\bar{c}}^{2}=\sigma_{a}^{2}/\sigma_{c}^{2},$ where $\sigma_{c}^{2}=\sigma_{b}^{2}+\sigma_{p}^{2}+\sigma_{a}^{2}+\sigma_{f}^{2}+\sigma_{c}^{2}+\sigma_{e}^{2}/r$, with *r* corresponding to the harmonic mean of the number of replicates of each genotype in each trial. The covariate of initial weight ***w*** was only measured and included in trials T1/2013 and T1/2014 for the traits where it was significant (evaluated by a Wald test using a significance level of 5%), and some of the other designs terms **b**(**r**) and **p**(**br**) were considered according to the design layout. Note that both term **b**(**r**) and **p**(**br**) were included to assist with control of spatial variability.

In order to evaluate genotype-by-environment interaction (G×E) for each of the traits, a model was fitted that combined the raw data from each pair of tests as:

(2)y=1µ+X1t+w(t)β+Z1r(t)+Z2b(rt)+Z3p(brt)+Z4a+Z5ta+Z6f+Z7tf+Z8c(f)+Z9tc(f)+e

where, *μ* is the overall mean; ***t*** is the fixed effect of trial; ***w***(***t***) is the covariate of initial weight of a plant within a trial; *r*(*t*) is a fixed effect of replicate within a trial; **b**(**rt**) is the random effect of bed within replicate and trial, with $b\left(\mathrm{rt}\right)∼MVN\left(0,\;\sigma_{b}^{2}D_{b}\right);$ **p**(**brt**) is the random effect of plot within a bed, replicate and trial, with $p(\mathrm{brt})∼MVN(0,\;\sigma_{p}^{2}D_{P}$); **a** is the random additive effect of a genotype, where $a∼MVN(0,\sigma_{a}^{2}A)$; **ta** is the random interaction effect of genotype with trial, with $ta∼MVN\left(0,\sigma_{ta}^{2}I\right)$; **f** is the random family effect, with $f∼MVN(0,\sigma_{f}^{2}I)$; **tf **is the random interaction effect of family with trial, with $tf∼MVN\left(0,\sigma_{tf}^{2}I\right)$; ***c***(***f***) is the random effect of clone within family, with $c(f)∼MVN(0,\sigma_{c}^{2}I);$ **tc**(**f**) is the random interaction effect of trial with clone within family, with $\;tc(f)∼MVN(0,\sigma_{tc}^{2}I)$; and **e** is the residual term, with $e∼MVN(0,\sigma_{e}^{2}D_{e})$. The matrices **D** corresponds to diagonal matrices that estimates a different variance component for each of the trials, and all other terms were previously defined. The G×E interaction due to both the interaction of additive effects between pairs of trials and the interaction of additive effects between pairs of trials in different seasons was estimated using Type-B genetic correlations,^24^ calculated as: $r_{B}^{2}=\sigma_{a}^{2}/(\sigma_{a}^{2}+\sigma_{ta}^{2})$. This genetic correlation varies from zero to one, with values near one indicating low G×E and similar rankings of genotypes across trials and/or years.

In order to obtain an adjusted phenotypic mean prediction, *y*_adj_, for each genotype to be used in the genomic prediction approaches, the following model was fitted for each of the trials and traits:

(3)y=1µ+wβ+Xr+Z1b(r)+Z2p(br)+Z3g+e

where all terms were random effects and were previously defined with the exception of **g** which corresponds to the total genetic value (that is, clonal value) and here it was assumed to be a fixed effect.

All of the previous models were fitted using ASReml v. 3.0,^25^ which estimates variance components based on residual maximum likelihood using the raw information available from the field trials. In all cases, residuals were checked to verify assumptions, and no important departures from normality were observed.

### Methods of genomic prediction

In order to evaluate the performance of GS, six contrasting methods were assessed in this study. The selected methods corresponded to: Bayes B,^11^ Bayes C,^12^ Bayes Ridge Regression, BRR,^26^ Bayesian LASSO, BL,^27^ Bayesian Reproducing Kernel Hilbert Spaces, RKHS^28^ and Genomic BLUP, GBLUP.^29^ The Bayesian methods address the problem of small number of observations (*n*) and a large number of parameters (*p*) to be estimated (*n*<<*p*) by restricting the size of the regression coefficients via shrinkage or regularization.^11^ The package BGLR^30^ implemented in the package R version 3.1.1(ref. 31) was used to estimate genomic predictions for Bayes B, Bayes C, BL, BRR and RKHS, with the response variables corresponding to the adjusted phenotypic mean values obtained previously (Equation 3).

The Bayesian models for continuous variables are represented by the equation **y**=**1***μ*+**Xβ**+**e**, where **y** is the vector of adjusted phenotypic observations, *μ* is an intercept, **β** is a vector of marker effects associated to the columns of the marker incidence matrix **X**, and **e** is the vector of residual effects. The conditional distribution of marker effects of these models differ in the allocation of priors, which determine the type of shrinkage or variable selection imposed on the estimates.^32^ In Bayes B and Bayes C, a mixture of two different finite prior densities is used, a point of mass at zero and a Gaussian slab for Bayes B and a point of mass at zero and a scaled-t slab in Bayes C. In BRR a Gaussian prior density gives similar shrinkage to all marker effects and in BL a double exponential prior density causes a size of effect-dependent shrinkage on the estimates.^32^ In this study, each of the different prior densities were defined by using the default hyper-parameters presented in Perez and de los Campos.^32^ In preliminary work, different parameters were evaluated but these did not show any important differences over the default recommended parameter values.

BGLR also implements RKHS regression which was proposed for the prediction of genetic values by Gianola^28^ and have been applied for complex traits in wheat breeding and animal breeding.^33^ This method uses a function or Reproducing Kernel (RK) that maps ‘marker genotypes $K(m_{i},m_{i\prime })$ from an input set onto a real line that must satisfy $\sum^{\;}i\;\sum^{\;}i\prime \alpha_{i}\alpha_{i\prime }K\left(m_{i},m_{i\prime }\right)\geq 0,\;\;$for any non-null sequence of coefficients *α*_*I*_’.^32^ In this method, the observed numerator relationship matrix (**A**_g_) is replaced by the kernel matrix (**K**), using a Gaussian prior evaluated by the square Euclidean distance between markers.^34^ The bandwidth parameter *h,* and the residual variance indexed by a scale and degrees of freedom parameters were set using default values.^32^

In GBLUP, an observed numerator relationships matrix, **A**_g_, was obtained using all 17 479 markers with no imputation for missing values. This matrix was calculated by using the equations described by Yang *et al.*,^35^ and later an inverse of this matrix was generated implementing bending due to the presence of non-positive eigenvalues.^36^ Markers quality control, generation of the **A**_g_ matrix and its inverse were all performed with the software GenoMatrix.^37^ This matrix was later used, based on the adjusted phenotypic values, to fit a simple animal/individual model of the form: **y**=**1***μ*+**Z*****a***+***e***, where $a∼MVN(0,\sigma_{a}^{2}A_{g})$ and $e∼MVN\left(0,\sigma_{e}^{2}I\right)$. Model fitting for GBLUP and pedigree-based analysis, PBLUP, together with the generation of genomic predictions for each of the genotypes was performed with the software ASReml-R^38^ as implemented for the statistical package R version 3.1.1.^31^

### Evaluation of GS methods

The assessment of the different GS methods, for each of the traits and trials, was done by calculating the predicted ability (PA) and the prediction accuracy (PACC) of the genomic estimated breeding values (GEBV). PA was estimated as the correlation between the adjusted phenotypic value and the GEBV, $corr(y_{adj},\hat{a})$, based on a given GS model. This was done by fitting the model with marker data and phenotypic data from a given trial as training population (for example, T1/2013), and predicting to itself by a fourfold cross-validation, and to other trials as a true validation (for example, T2/2013, T1/2014, T2/2014). Cross-validation was performed by randomly selecting four-fifths of the individuals for the training population and the remaining fifth as the validation population, repeating the procedure until all individuals in the trial were validated. Cross-validations are expected to generate higher PA and PACC than true validations given that training and validation datasets are the same, which increases the risk of model overfitting.

The PACC of GEBV, or the correlation between the true genomic breeding value and the predicted breeding value,$corr(a,\hat{a})$, for a specific GS model, was estimated as: $PACC=PA/\sqrt{h_{\bar{c}}^{2}}$ where $h_{\bar{c}}^{2}$ is the *ad hoc* heritability described earlier. For PACC comparisons, a PBLUP analysis was performed, that replaces the genomic relationship matrix (**A**_g_) from the marker data with its traditional counterpart (**A**) obtained from pedigree information.

### Genetic map and linkage disequilibrium (LD) estimation

JoinMap 4.1 software^39^ was used to create a high-density SNP linkage map of 14 332 SNP markers using a FL_08-10×12.115-10 mapping population comprised of 165 progeny (Verma *et al*.,^18^ unpublished data). Because of stringent mapping parameters and in order to minimize gaps between SNPs, a few linkage groups (LG) were subdivided into two groups. Orientation and subgenome specificity of each LG were assigned according to van Dijk *et al.*^40^ In order to evaluate the contribution of LD and genetic relationships on the accuracy and efficiency of GS methods, LD parameters were calculated for the entire T2/2013 and T2/2014 population sets. Common SNPs between the mapped markers and the 17 479 SNPs chosen for the genomic analysis were extracted. A common set of 4841 SNPs were distributed in 28 LGs and analyzed for extent of LD. Pairwise LD (*r*^2^) for each LG was estimated using the R package LDheatmap.^41^ The genomic relationship matrix derived from each LG was estimated using GenoMatrix software^37^ and used for the estimation of LD corrected for relatedness $\left(r_{v}^{2}\right)$ utilizing the LDcorSV package in R.^42^

### Selection efficiency

Parent selection efficiency was used to experimentally evaluate the performance of PBLUP, GBLUP and Bayes B versus indirect selection in a true validation context. This consisted of calculating the ratio of genetic gain when selecting the top 5 and 10% genotypes obtained by using incomplete information versus complete information for selection. The incomplete information scenario consisted of using a GS model trained in trial T2/2013 to predict genetic values in trial T2/2014, using only marker data from T2/2014. This represents a typical GS strategy in which phenotypes have not yet been obtained. In the complete information scenario the predicted genetic values from the trial T2/2014 were calculated using both the phenotypic and marker information from T2/2014 by fitting a GBLUP model. This represents best available analysis practice given that both phenotypic and marker data T2/2014 were available for ranking and selection. In theory, a perfect GS model would achieve 100% of the genetic gain (100% selection efficiency) achieved in the complete information scenario.

## Results

### Quantitative genetic parameters

A summary of the phenotypic data are presented for each of the trials and traits (Supplementary Table S1). In general, there was a wide range of phenotypic variability for each trial and trait under study. For the single-site analysis of each of the trials based on the model from Equation 1, moderate to low levels of narrow-sense heritability values (*h*^2^) were found (Table 2). However, there are important differences between trials, where T2/2014 had some of the lowest heritability values. The highest average *h*^2^ across trials was found for AWT (average of 0.38), and the lowest levels were detected for TMY and EMY (averages of 0.17 and 0.18, respectively). Broad-sense heritability (*H*^2^) averages were high (0.62 for AWT) to moderate (0.38–0.47) for the remaining traits. Non-additive effects varied on average across trials and years between 0.20 for EMY to 0.27 for TMY, however, there was a high range of variability of non-additive genetic effects between different combinations of traits and trials.

Type-B additive genetic correlation estimates showed a wide range of G×E interactions, with no interaction for AWT and SSC, low to moderate for TC and high for EMY and TMY (Supplementary Table S2). The correlations of importance for the true validation of GS methods are between either T1/2013 or T2/2013 with both T1/2014 and T2/2014. High levels of G×E were found for T1/2013 with both T1/2014 and T2/2014 for EMY and TMY, but low levels of G×E were found between trials T2/2013 and T2/2014, with a moderate level for EMY. These levels of G×E are critical in the case that T2/2013 is used as a training dataset and T2/2014 as validation dataset with the implementation of the genomic selection methods, where G×E effects are not predicted and are therefore absorbed by the residual error.

### Comparison of methods of genomic prediction

**Figure 1:**
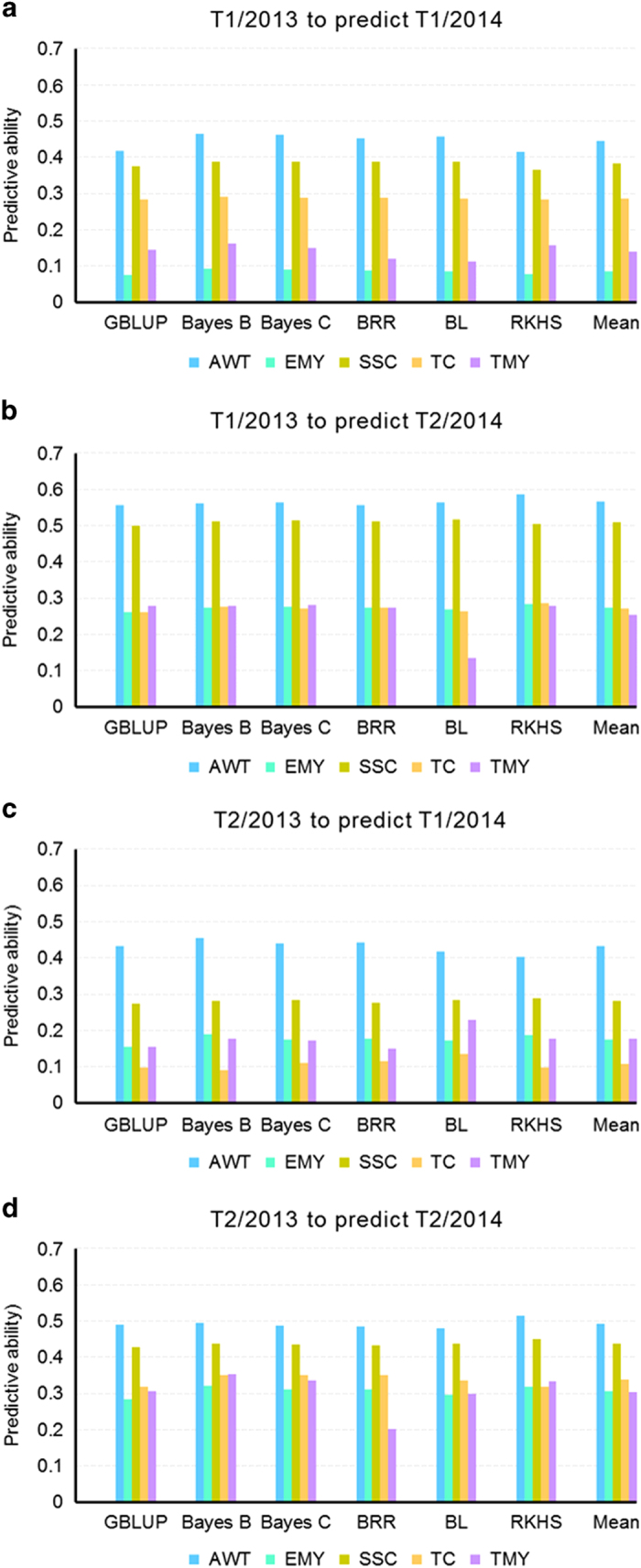
Predictive ability (PA) for true validation of several genomic selection (GS) methods, based on a model fitted in either T1/2013 or T2/2013 to predict both T1/2014 and T2/2014. The mean corresponds to the average of GS methods. (**a**) model fitted in T1/2013 to predict T1/2014, (**b**) model fitted in T1/2013 to predict T2/2014, (**c**) model fitted in T2/2013 to predict T1/2014, and (**d**) model fitted in T2/2013 to predict T2/2014.

True validations, obtained by fitting a GS model from one trial and evaluating the model in a different trial, resulted in a wide range of PA varying from 0.08 to 0.59 (Figure 1). Even though the training population sizes for T1/2013 and T2/2013 were different (647 vs 244), the average PA across the five traits were similar (0.24 and 0.27) to predict T1/2014 and also similar when both 2013 trials were used to predict T2/2014 (0.38). For the model fitted in T1/2013 to predict T1/2014, Bayes B exhibited the highest PA for all traits, while GBLUP and RKHS were the lowest. For the model fitted in T1/2013 to predict T2/2014, GBLUP again had the lowest PA for most traits, whereas RKHS performed much better, even higher than Bayes B for some traits. In general, Bayes B, closely followed by RKHS, gave the best predictive abilities across all traits, with average PAs of 0.323 and 0.317, respectively; whereas GBLUP had an average PA of 0.305, and Bayes C, BRR and BL gave intermediate values.

Of special interest are the models built for T1/2013 and T2/2013 to predict T2/2014, due to their higher PA estimates compared with the other two models presented in Figure 1. For those two models the average trait PA ranged from 0.27 to 0.57 and from 0.31 to 0.49, respectively. The model fitted in T1/2013 to predict T2/2014 follows the current path of the breeding cycle, where some selections from T1/2013 are established the following year. However, the model fitted in T2/2013 to predict T2/2014 had the second highest PA correlations and, importantly, the best PA for yield traits. The correlation between average PA and heritability was high (0.79) for the model fitted in T1/2013 to predict T2/2014, with a lower correlation (0.55) for the model fitted in T2/2013 to predict T2/2014.

Results of the fivefold cross-validation within each trial for all GS methods are included in the Supplementary Materials (Supplementary Table S3), but partial results of cross-validations within each trial for methods with the highest PA (Bayes B and RKHS) and lowest PA (GBLUP) are presented in Table 3. Cross-validation PA values were, in most cases, higher for all GS models when compared with those from true validation except for SCC in T1/2013 and TMY in T2/2013. Here, RKHS and GBLUP had the highest cross-validation PAs in all trials for most traits, with RKHS showing the highest PA in trials established in 2013 and GBLUP in the 2014 trials (Supplementary Table S3). These results are in contrast with those for true validation which used independent testing populations.

Prediction accuracies calculated from the true validation models had average values ranging from 0.37 to 0.75 across the GS methods with largest average values for the traits SSC and TMY, and lowest for TC (Table 4). The prediction accuracy for the traditional pedigree-based analysis (PBLUP) showed lower values for all traits compared with all GS methods, with an average predictive accuracy of 0.45 across traits, in contrast to 0.59 for the average of the GS methods, a clear indication of the advantages of molecular data to perform predictions. The trait that presented the lowest prediction accuracy, TC, was particularly poor for PBLUP (0.16).

### Linkage disequilibrium (LD)

**Figure 2:**
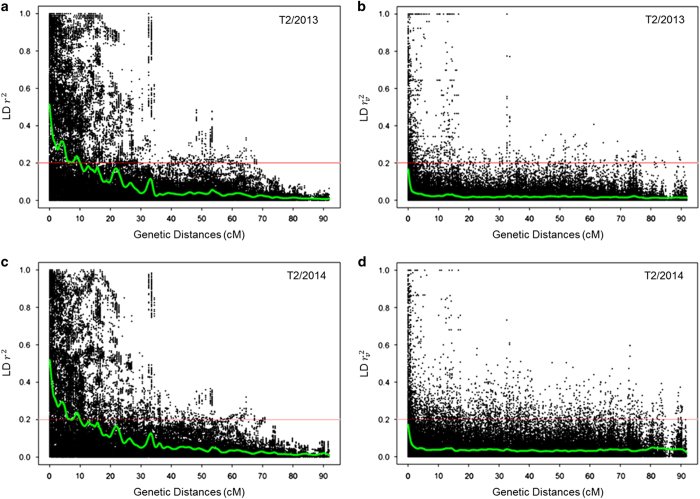
Scatter plot of pairwise regular linkage disequilibrium (LD) (*r*^2^) and LD corrected for relatedness $\left(r_{v}^{2}\right)$ against increasing genetic distances between markers on the LG 6A. (**a**) pairwise regular LD (*r*^2^) for individuals tested in the T2/2013 trial, (**b**) LD corrected for relatedness $\left(r_{v}^{2}\right)$ for the T2/2013 trial, (**c**) pairwise regular LD (*r*^2^) for the T2/2014 trial, and (**d**) LD corrected for relatedness $\left(r_{v}^{2}\right)$ over increasing genetic distances of LG 6A for the T2/2014 trial. Green lines indicate smoothed splines.

A set of 4841 SNPs, common between 17 479 polymorphic SNPs chosen for genomic selection analysis and 14 332 genetically mapped SNPs, were distributed in 28 LGs (Supplementary Table S4). The overall length of the genetic map was 1695.46 cM. For T2/2013, the average intra-linkage group regular pairwise LD (*r*^2^) was 0.26 and LD corrected for relatedness $\left(r_{v}^{2}\right)$ was 0.04. For T2/2014, the average intra-linkage group regular pairwise LD (*r*^2^) was 0.26 and LD corrected for relatedness $\left(r_{v}^{2}\right)$ was 0.05. Thus, overall pairwise regular LD (*r*^2^) declined significantly after correcting for relatedness $\left(r_{v}^{2}\right)$ (Figure 2). For each LG, as the genetic distance increased both *r*^2^ and $r_{v}^{2}$ decreased but *r*^2^ decreased at a slower rate than $r_{v}^{2}$ (Figure 2 for LG 6A). The extent of LD (*r*^2^) was >0.2 until 10 cM, however, LD $\left(r_{v}^{2}\right)$dropped to ~0.05 within ~2 cM for LG 6A (Figure 2). The *r*^2^, $r_{v}^{2}$, and pattern of LD decay for T2/2014 were similar to T2/2013 (Supplementary Figures S1 and S2).

### Selection efficiency

**Figure 3:**
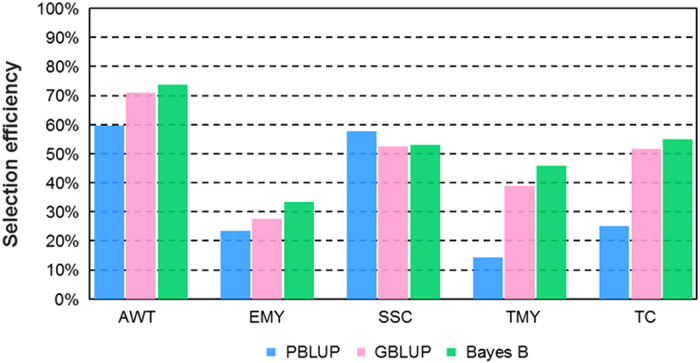
Selection efficiency based on genetic gains when selecting the top 10% of genotypes (corresponding to 30 individuals) for three different GS models fitted with T2/2013 phenotypic and marker data for training and used to make predictions in T2/2014 using T2/2014 marker data only. Gains are compared with breeding values for T2/2014 estimated using both phenotypic and marker data from T2/2014. AWT, average weight (g per fruit); EMY, early marketable yield (g per plant); SSC, soluble solids content (%); TC, proportion of total culls (%); TMY, total marketable yield (g per plant).

The efficiency of parent selection for each trait, expressed as a function of genetic gains, and obtained by performing selection of the best 5 and 10% of genotypes using the complete information scenario (combining the phenotypic information with molecular data to select the highest performing genotypes in T2/2014 in terms of breeding value), against the incomplete information scenario (genetic predictions from a model fitted with T2/2013 but predicting genotypic performance in T2/2014 using markers only) is presented in Figure 3 and Supplementary Table S5. We would expect 100% efficiency in terms of genetic gain if all individuals selected (top 10%) in T2/2014 using a model trained in T2/2013 are exactly the same as those selected using both phenotypic and molecular data from T2/2014. This figure indicates that, for some traits, efficiencies above 50% can be achieved with the use of predictions from GS models fitted in T2/2013 based on incomplete information. The lowest values were found, as expected, using PBLUP and for the lowest heritability traits EMY and TMY. The differences in efficiency of selection between the GS methods GBLUP and Bayes B were smaller, with Bayes B giving an average selection efficiency across traits of 52.2% vs 48.3% for GBLUP, and 36.0% for PBLUP.

## Discussion

The main objective of the present study was to evaluate GS methods in octoploid strawberry, with the practical aim of incorporating the GS in strawberry breeding, using the University of Florida strawberry breeding program as an example. The magnitudes of PA, PACC and selection efficiency obtained for five complex traits indicate that genomic selection is a promising tool for genetic improvement of quantitative traits in strawberry. In particular, high prediction accuracies and selection efficiencies from true validations, using independent trials from two consecutive years, strongly support the potential utility of GS in a practical strawberry breeding context. In general, our PA estimates are consistent in magnitude with other studies using true validation, as is the case in apple,^8^ sugarcane^43^ and bread wheat.^44^

For cross-validations within training populations, predictive abilities were higher than for true validations across trials/years, apparently due to overfitting. Several studies have shown that the prediction accuracy decreases when cross-validation within the same training population is compared with true validation based on independent trials.^45,46^ Furthermore, based on cross-validation, RKHS or GBLUP would appear to be the best choices for the application of GS. In contrast, for true validations Bayes B gave the highest average PA and the highest average selection efficiency, and performed particularly well for the lowest heritability traits (EMY and TMY) for which genetic gain is at a premium. The cross-validation PA estimates of this study are within the range of other plant and tree studies, as reported for tomato,^16^ bread wheat^44^ and Maritime pine.^47^ PACC estimates of SSC using cross validation reported for tomato,^16^ sugarcane^43^ and sugar beet^48^ are smaller than our estimates for the same trait using true validation. However, larger estimates of PACC for SSC were reported in an apple study (PA=0.86) compared with the present study, possibly due to higher LD (*r*^2^=0.32) in the studied population.^14^

As the models evaluated in this study are based on prediction of additive genetic effects, parent selection and selection of cross-combinations are of the most immediate relevance. However, strawberry cultivars are deployed as clones, and the prediction of non-additive genetic effects would be of great value in the future for predicting clonal performance. Indeed, the high proportions of non-additive variance for all traits under study (Table 2) indicates the need to fit GS models that account for non-additive genetic effects in the future. Some univariate genetic models with non-additive effects^46,49^ have shown small to moderate improvements in prediction accuracy and bias reduction. In addition, a recent study of GS on rice hybrids using univariate and multivariate models indicated that, in single trait models, PA is improved for some traits by including dominance effects.^50^

On average the Bayes B method had higher PA and PACC than the other methods for all traits and trials in the present study, and was superior to GBLUP. This was particularly the case for the yield traits EMY and TMY. However, the difference of PACC estimates for AWT and SSC between Bayes B and GBLUP were very small, indicating that the methods perform equivalently for these traits in the T2/2013 and T2/2014 populations. For some trials and traits, RKHS gave similar results to Bayes B. RKHS is a method that has shown better results that other methods in some studies, and its superiority is attributed to the capture of some non-additive effects.^28,51^ In a simulation study in Asian rice, RKHS performed better than GBLUP, Bayesian methods and other non-parametric methods when traits had high heritability, presence of epistasis and were controlled by a large number of QTLs.^52^ In a simulation study comparing PBLUP, Bayes B and GBLUP under different genetic models (that is, major QTL model, rare variant model and the infinitesimal model), Bayes B showed higher accuracy of breeding values for the QTL and rare variant models and similar accuracy for the infinitesimal model.^53^ In a simulation study in barley, under a high-density marker scenario, two Bayes B models with different priors (*π*) had better prediction accuracies when the trait was controlled by less than 20 QTL, than RR-BLUP and a BLUP model based on the marker relationship matrix.^54^ Moreover, the authors concluded that the BLUP models mainly capture genetic relationships, whereas the Bayesian models captured both genetic relationships and marker-QTL associations based on LD.

These previous studies suggest that the moderate difference (0–15%) in PACC for Bayes B over GBLUP in the present study may be due to the better capture of QTLs in LD with markers, particularly for the yield traits EMY and TMY (Table 4). On the other hand, genetic relatedness might be just as important as LD for traits such as AWT and SSC. The fact that PBLUP had some value for indirect selection in this study, particularly for AWT and SSC, is indicative of the strong pedigree connectivity within the main breeding population at the University of Florida (Figure 3). This is not surprising, as this main or ‘elite’ breeding population results from over twenty generations of phenotypic recurrent selection. The LD estimates from both T2/2013 and T2/2014 indicate strong pairwise marker associations, even at distances of 10 cM or more and even when correcting for genetic relatedness (Figure 2). Although differences in marker density across LGs resulted in variability between *r*^2^ and$r_{v}^{2}$ among LGs (Supplementary Table S4), the average marker density across the genome was 2.86 marker per cM, which is adequate for genome-wide LD estimation.^14,46^ For comparison, the genome-wide estimates of LD for T2/2013 (*r*^2^=0.26 and $r_{v}^{2}=0.04$) are much larger than for maritime pine ($r_{v}^{2}=0.006$ and *r*^2^=0.011),^47^ but slightly less than apple (*Malus*×*domestica* Borkh.; *r*^2^=0.32).^14^ Although some studies have been designed to partition observed prediction accuracy into the portion due to LD and the portion due to genetic relatedness,^54–57^ the present study was not designed to allow such a partitioning.

From a breeding standpoint, it is noteworthy that predictive abilities for the true validation from T2/2013 to T2/2014 were nearly as high on average as those from T1/2013 to T2/2014. The predictions from both T1/2013 and T2/2013 to T2/2014 are important for selecting parents because T2 populations contain the parent pool for future crossing. Besides, it is expected that the PA and PACC will increase as the population size of the T2 training populations is augmented by combining marker data from successive cycles of breeding. Several authors have shown the impact of increasing the size of the training population and improving the genetic relationships between training and testing populations on the estimation of prediction accuracy.^4,7^ Both T1/2013 and T2/2013 showed similar average PA across all traits when predicting T2/2014 despite the fact that both shared a similar number of parents, 29 and 40, respectively, with T2/2014 but had different population sizes (647 vs 244). This discrepancy might be explained by the fact that the T1 populations are seedling populations from mating designs of about 60 crosses, plus controls and other specific complementary crosses, and T2 is derived from the T1 trials, plus advanced selections from previous T2 trials with lower genetic diversity than T2. This disparity between trials reduces prediction ability in T1 trials due to more genotypes from different crosses and increases PA in T2 due to a higher ratio of genetic relationships between T2/2013 and T2/2014.

**Figure 4:**
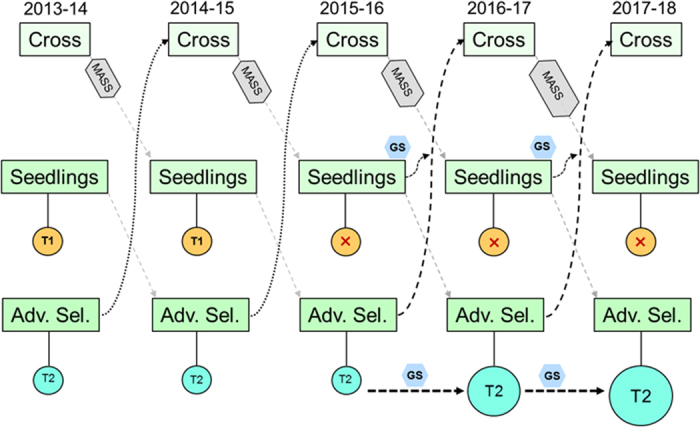
Breeding strategy transition to an approach combining marker-assisted seedling selection (MASS) for major loci and genomic selection (GS) as an early parent selection method for complext traits. The incorporation of GS for parent selection allows the use of first-year seedlings in crosses prior to their inclusion in a replicated advanced selection trials (T2). Trials of unselected seedlings (T1) will be eliminated in favor of increasing the number of genotypes evaluated in T2 trials, which will be used as training populations for future GS models.

The T2 trials also have added practical value in that these trials are essential for phenotypic testing of advanced selection performance as clones. On the other hand, the T1 trials are comprised mainly of unselected seedlings, and their main value lies in estimation of genetic parameters and parental breeding values. Thus, these results suggest that a cost savings could be obtained by eliminating yearly T1 replicated trials and focusing efforts on T2 replicated trials for both phenotypic testing of clones and training GS models for future parent selection. This is what we propose for the University of Florida strawberry breeding program (Figure 4). By focusing effort on T2 trials, perhaps including a greater number of genotypes and measuring additional traits, resources can be used in a more focused manner. Here seedlings can be predicted for parental performance one year prior to their inclusion in the next T2 trial. Thus, some genotypes that are predicted by GS to be the best parents for a combination of traits can be immediately selected as parents. Under this scenario, the breeding cycle can be reduced from 3 to 2 years for some selections. Our study has shown that efficiencies from early selection are not optimal (Figure 3); however, they provide relevant genetic gains at an earlier time without requiring the phenotyping of the individuals. For example, by using Bayes B for average weight, it is possible to achieve ~70% of the optimal genetic gain 1 year earlier.

In summary, where both phenotypic and marker data are available in strawberry breeding populations, GS models should allow more precise breeding value estimates compared with models utilizing only pedigree information. In addition, with appropriate statistical models such as Bayes B, parent selection efficiencies near to and even >50% can be obtained for several commercial traits when phenotypic information is not yet available. For some parents predicted early to be top performers, this methodology may allow a reduction of the breeding cycle from 3 to 2 years, with the goal of increasing genetic gains over time for several complex yield and fruit quality traits. The results also suggest that replicated advanced selection trials have potential as training populations for predicting parental performance in the next cycle of breeding. In the future, we plan to expand this study to the prediction of clonal performance, as well as the potential of GS for seedling selection within full-sib families, should a reduction in genotyping costs make this approach economically feasible in strawberry.

## Figures and Tables

**Table 1:** Number of parents (represented by their progeny) and individuals tested among four field trials

*Trial*	*T1/2013*	*T2/2013*	*T1/2014*	*T2/2014*
T1/2013	41/647	10	5	39
T2/2013	28	86/244	7	39
T1/2014	14	14	45/610	5
T2/2014	29	40	20	64/333

The diagonal indicates the total number of parents (first value) followed by the number of cultivars or advanced selections in each trial (second value). Below the diagonal are the common parents represented between pairs of trials, and above the diagonal are individuals (cultivars or advanced selections) represented between pairs of trials.

**Table 2:** Single-trial narrow-sense heritability (*h*^2^) and broad-sense heritability (*H*^2^) based on the original observations

*Trial*	*AWT*		*EMY*	
	h*^2^*	H*^2^*	h*^2^*	H*^2^*
T1/2013	0.42 (0.10)	0.60 (0.03)	0.35 (0.10)	0.43 (0.04)
T2/2013	0.46 (0.12)	0.62 (0.04)	0.11 (0.07)	0.29 (0.04)
T1/2014	0.38 (0.10)	0.66 (0.03)	0.24 (0.08)	0.42 (0.03)
T2/2014	0.25 (0.12)	0.59 (0.03)	0.03 (0.07)	0.39 (0.04)
		
	SSC		TC	
T1/2013	0.35 (0.09)	0.38 (0.04)	0.26 (0.10)	0.38 (0.04)
T2/2013	0.18 (0.08)	0.34 (0.04)	0.44 (0.06)	0.47 (0.04)
T1/2014	0.15 (0.06)	0.39 (0.03)	0.18 (0.07)	0.48 (0.03)
T2/2014	0.41 (0.10)	0.46 (0.04)	0.08 (0.12)	0.53 (0.03)
			
	TMY			
T1/2013	0.21 (0.09)	0.46 (0.04)		
T2/2013	0.11 (0.10)	0.38 (0.04)		
T1/2014	0.11 (0.08)	0.44 (0.03)		
T2/2014	0.26 (0.10)	0.47 (0.04)		

Abbreviations: AWT, average weight (g per fruit); EMY, early marketable yield (g per plant); SSC, soluble solids content (%); TC, proportion of total culls (%); TMY, total marketable yield (g per plant).

Approximated s.e. are presented in parentheses.

**Table 3:** Predictive ability (PA) from fivefold cross-validation for three different GS models built in T1/2013 and T2/2013

*Model*	*T1/2013*			*T2/2013*		
	*GBLUP*	*Bayes B*	*RKHS*	*GBLUP*	*Bayes B*	*RKHS*
AWT	0.599	0.598	0.600	0.526	0.532	0.538
EMY	0.489	0.480	0.500	0.314	0.292	0.341
SSC	0.440	0.428	0.434	0.532	0.513	0.561
TC	0.285	0.280	0.308	0.492	0.483	0.532
TMY	0.444	0.422	0.462	0.203	0.177	0.257

Abbreviations: AWT, average weight (g/fruit); EMY, early marketable yield (g/plant); SSC, soluble solids content (%); TC, proportion of total culls (%); TMY, total marketable yield (g/plant).

**Table 4:** Prediction accuracy (PACC) for different GS methods based on a model fitted in T2/2013 to predict T2/2014

*Trait*	*PBLUP*	*GBLUP*	*Bayes B*	*Bayes C*	*BRR*	*BL*	*RKHS*	*Mean*	$h_{\bar{c}}^{2}$
AWT	0.549	0.606	0.610	0.603	0.601	0.594	0.636	0.608	0.655
EMY	0.415	0.557	0.629	0.608	0.608	0.580	0.621	0.600	0.262
SSC	0.630	0.708	0.726	0.723	0.719	0.726	0.748	0.725	0.364
TC	0.159	0.365	0.400	0.402	0.401	0.385	0.363	0.386	0.767
TMY	0.507	0.652	0.753	0.718	0.433	0.638	0.710	0.630	0.220

Abbreviations: AWT, average weight (g/fruit); EMY, early marketable yield (g/plant); SSC, soluble solids content (%); TC, proportion of total culls (%); TMY, total marketable yield (g/plant).

The mean corresponds to the average of GS methods and $h_{\bar{c}}^{2}$ is the *ad hoc* heritability of T2/2013.
