# Supplementary material for: An experimental validation of genomic selection in octoploid strawberry
Source: Hortic Res. 2017 Jan 11;4:16070–. doi: 10.1038/hortres.2016.70 (PMC5225750; doi:10.1038/hortres.2016.70)
Supplement: Supplementary Information [file hortres201670-s1.doc]

An experimental validation of genomic selection in octoploid strawberry

Salvador A. Gezan^1^, Luis F. Osorio^2^, Sujeet Verma^2^ and Vance M. Whitaker^2^

^1^ University of Florida, School of Forest Resources and Conservation, 363 Newins-Ziegler Hall, PO Box 110410, Gainesville, Florida 32611-0410, USA.

^2^  University of Florida, Gulf Coast Research and Education Center, 14625 CR 672, Wimauma, Florida 33598, USA

Corresponding author**:** Vance M. Whitaker Email: [vwhitaker@ufl.edu](mailto:vwhitaker@ufl.edu)

This supplementary materials file totaling nine pages contains five supplementary tables, S1-S5, followed by two supplementary figures, S1-S2.

**SUPPLEMENTARY TABLES**

**Supplementary Table S1.** Summary statistics (number of individuals tested, mean and range) for each trial and trait under study. AWT: average weight (g/fruit), EMY: early marketable yield (g/plant), SSC: soluble solids content (%), TC: proportion of total culls (%), TMY: total marketable yield (g/plant)

| Trial |  | N |  | Mean (Range) | | | | | | | | |
| --- | --- | --- | --- | --- | --- | --- | --- | --- | --- | --- | --- | --- |
|  |  |  |  | AWT |  | EMY |  | SSC |  | TC |  | TMY |
| T1/2013 |  | 647 |  | 19.5 |  | 177.7 |  | 7.4 |  | 41.4 |  | 449.2 |
|  |  |  |  | (10.0-37.3) |  | (10-813) |  | (4.0-13.0) |  | (14.1-94.2) |  | (10-1,305) |
| T2/2013 |  | 244 |  | 19.4 |  | 238.7 |  | 7.3 |  | 27.9 |  | 522.3 |
|  |  |  |  | (11.0-32.7) |  | (10-782) |  | (3.6-13.7) |  | (8.0-79.0) |  | (11-1,369) |
| T1/2014 |  | 610 |  | 23.8 |  | 297.0 |  | 7.6 |  | 32.0 |  | 539.3 |
|  |  |  |  | (12.7-43.1) |  | (10-701) |  | (3.7-12.1) |  | (7.6-99.9) |  | (10-1,201) |
| T2/2014 |  | 333 |  | 25.0 |  | 284.0 |  | 7.9 |  | 25.2 |  | 615.9 |
|  |  |  |  | (12.0-43.6) |  | (10-749) |  | (5.0-11.8) |  | (6.7-99.5) |  | (12-1,552) |

**Supplementary Table S2.** Type-B additive genetic correlations between pairs of trials obtained by fitting Eq.2. Approximated standard errors, based on delta method, are presented in parentheses. AWT: average weight (g/fruit), EMY: early marketable yield (g/plant), SSC: soluble solids content (%), TC: proportion of total culls (%), TMY: total marketable yield (g/plant).

| Trait |  |  | AWT^1^ | | | |
| --- | --- | --- | --- | --- | --- | --- |
|  |  | Trial | T1/2013 | T2/2013 | T1/2014 | T2/2014 |
| EMY |  | T1/2013 | - | 0.63 (0.33) | 0.00 (0.00) | 0.65 (0.24) |
|  |  | T2/2013 | 0.95 (0.17) | - | 0.78 (0.04) | 0.74 (0.02) |
|  |  | T1/2014 | 0.96 (0.15) | 1.00 (0.00) | - | 1.00 (0.00) |
|  |  | T2/2014 | 0.98 (0.06) | 1.00 (0.00) | 0.88 (0.13) | - |
|  |  |  | SSC | | | |
|  |  | Trial | T1/2013 | T2/2013 | T1/2014 | T2/2014 |
| TC |  | T1/2013 | - | 0.72 (0.16) | 0.99 (0.10) | 0.86 (0.13) |
|  |  | T2/2013 | 1.00 (0.00) | - | 0.80 (0.18) | 1.00 (0.00) |
|  |  | T1/2014 | 0.91 (0.26) | 1.00 (0.00) | - | 1.00 (0.00) |
|  |  | T2/2014 | 0.98 (0.04) | 1.00 (0.00) | 1.00 (0.00) | - |
|  |  |  | TMY | | | |
|  |  | Trial | T1/2013 | T2/2013 | T1/2014 | T2/2014 |
|  |  | T1/2013 | - |  |  |  |
|  |  | T2/2013 | 0.81 (0.19) | - |  |  |
|  |  | T1/2014 | 0.00 (0.00) | 0.75 (0.23) | - |  |
|  |  | T2/2014 | 0.50 (0.22) | 1.00 (0.00) | 1.00 (0.00) | - |

^1^ AWT, SSC and TMY correlations are below the diagonal. EMY and TC are above the diagonal.

**Supplementary Table S3.** Predictive ability (PA), correlations between observed and predicted additive genotypic values for all traits evaluated based on a 5-fold cross-validation using GBLUP, Bayes B and RKHS, using the same sets for validation/training in all GS methods. For Bayes B and RKHS a total of 15000 iterations where the first 5000 were discarded. AWT: average weight (g/fruit), EMY: early marketable yield (g/plant), SSC: soluble solids content (%), TC: proportion of total culls (%), TMY: total marketable yield (g/plant).

| Trait |  | Model |  | T1/2013 |  | T2/2013 |  | T1/2014 |  | T2/2014 |
| --- | --- | --- | --- | --- | --- | --- | --- | --- | --- | --- |
|  |  | GBLUP |  | 0.599 |  | 0.526 |  | 0.605 |  | 0.437 |
| AWT |  | Bayes B |  | 0.598 |  | 0.532 |  | 0.596 |  | 0.425 |
|  |  | RKHS |  | 0.600 |  | 0.538 |  | 0.580 |  | 0.436 |
|  |  | GBLUP |  | 0.489 |  | 0.314 |  | 0.355 |  | 0.428 |
| EMY |  | Bayes B |  | 0.480 |  | 0.292 |  | 0.353 |  | 0.421 |
|  |  | RKHS |  | 0.500 |  | 0.341 |  | 0.353 |  | 0.432 |
|  |  | GBLUP |  | 0.440 |  | 0.532 |  | 0.438 |  | 0.473 |
| SCC |  | Bayes B |  | 0.428 |  | 0.513 |  | 0.431 |  | 0.452 |
|  |  | RKHS |  | 0.434 |  | 0.561 |  | 0.431 |  | 0.438 |
|  |  | GBLUP |  | 0.285 |  | 0.492 |  | 0.316 |  | 0.607 |
| TC |  | Bayes B |  | 0.280 |  | 0.483 |  | 0.311 |  | 0.576 |
|  |  | RKHS |  | 0.308 |  | 0.532 |  | 0.304 |  | 0.599 |
|  |  | GBLUP |  | 0.444 |  | 0.203 |  | 0.311 |  | 0.448 |
| TMY |  | Bayes B |  | 0.422 |  | 0.177 |  | 0.284 |  | 0.442 |
|  |  | RKHS |  | 0.462 |  | 0.257 |  | 0.318 |  | 0.411 |

**Supplementary Table S4.** Summary statistics of regular linkage disequilibrium (
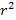
) associated with each linkage group (LG) and LD after correcting for relatedness (
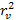
) among individuals of T2/2013. In the last row italic numbers represent the total sum of the number of markers and length of the genome, as well as the average value of the
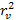
 statistics.

|  |  |  |  | 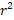 |  |  | 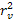 |  |  |
| --- | --- | --- | --- | --- | --- | --- | --- | --- | --- |
| Sub-LGs | LGs | no. of markers | Length (cM) | Mean | Minimum | Maximum | Mean | Minimum | Maximum |
| 1A | 1 | 95 | 9.88 | 0.26 | 0.00 | 1.00 | 0.05 | 0.00 | 1.00 |
| 1AII | 1 | 301 | 11.71 | 0.29 | 0.00 | 1.00 | 0.04 | 0.00 | 1.00 |
| 1B | 2 | 83 | 66.95 | 0.13 | 0.00 | 1.00 | 0.02 | 0.00 | 1.00 |
| 1C | 3 | 15 | 0.00 | 0.88 | 0.59 | 1.00 | 0.17 | 0.00 | 1.00 |
| 1CII | 3 | 49 | 20.02 | 0.24 | 0.00 | 1.00 | 0.01 | 0.00 | 1.00 |
| 1D | 4 | 7 | 16.76 | 0.29 | 0.00 | 0.81 | 0.00 | 0.00 | 0.00 |
| 1DII | 4 | 15 | 6.13 | 0.22 | 0.00 | 1.00 | 0.01 | 0.00 | 1.00 |
| 2A | 5 | 52 | 9.49 | 0.48 | 0.00 | 1.00 | 0.05 | 0.00 | 1.00 |
| 2AII | 5 | 89 | 12.62 | 0.27 | 0.00 | 1.00 | 0.04 | 0.00 | 1.00 |
| 2B | 6 | 59 | 12.87 | 0.53 | 0.00 | 1.00 | 0.02 | 0.00 | 1.00 |
| 2BII | 6 | 31 | 10.83 | 0.53 | 0.00 | 1.00 | 0.02 | 0.00 | 1.00 |
| 2C | 7 | 66 | 15.36 | 0.25 | 0.00 | 1.00 | 0.03 | 0.00 | 1.00 |
| 2CII | 7 | 58 | 26.64 | 0.21 | 0.00 | 1.00 | 0.04 | 0.00 | 1.00 |
| 2D | 8 | 25 | 2.44 | 0.52 | 0.00 | 1.00 | 0.03 | 0.00 | 1.00 |
| 3A | 9 | 152 | 24.46 | 0.25 | 0.00 | 1.00 | 0.03 | 0.00 | 1.00 |
| 3AII | 9 | 106 | 14.19 | 0.20 | 0.00 | 1.00 | 0.04 | 0.00 | 1.00 |
| 3B | 10 | 174 | 61.85 | 0.15 | 0.00 | 1.00 | 0.03 | 0.00 | 1.00 |
| 3C | 11 | 92 | 36.22 | 0.17 | 0.00 | 1.00 | 0.07 | 0.00 | 1.00 |
| 3CII | 11 | 164 | 57.01 | 0.27 | 0.00 | 1.00 | 0.08 | 0.00 | 1.00 |
| 3D | 12 | 302 | 136.12 | 0.07 | 0.00 | 1.00 | 0.02 | 0.00 | 1.00 |
| 4A | 13 | 28 | 14.20 | 0.65 | 0.23 | 0.98 | 0.06 | 0.00 | 1.00 |
| 4AII | 13 | 4 | 5.95 | 0.19 | 0.00 | 0.83 | 0.11 | 0.07 | 0.15 |
| 4B | 14 | 20 | 17.42 | 0.44 | 0.00 | 1.00 | 0.07 | 0.00 | 1.00 |
| 4BII | 14 | 58 | 28.03 | 0.15 | 0.00 | 1.00 | 0.04 | 0.00 | 1.00 |
| 4C | 15 | 78 | 73.53 | 0.11 | 0.00 | 1.00 | 0.04 | 0.00 | 1.00 |
| 4D | 16 | 71 | 46.19 | 0.14 | 0.00 | 1.00 | 0.17 | 0.00 | 1.00 |
| 5A | 17 | 205 | 50.14 | 0.13 | 0.00 | 1.00 | 0.03 | 0.00 | 1.00 |
| 5B | 18 | 25 | 13.51 | 0.24 | 0.00 | 0.99 | 0.01 | 0.00 | 0.87 |
| 5BII | 18 | 110 | 17.21 | 0.27 | 0.00 | 1.00 | 0.03 | 0.00 | 1.00 |
| 5C | 19 | 254 | 132.46 | 0.09 | 0.00 | 1.00 | 0.02 | 0.00 | 1.00 |
| 5D | 20 | 264 | 99.26 | 0.09 | 0.00 | 1.00 | 0.03 | 0.00 | 1.00 |
| 6A | 21 | 460 | 91.84 | 0.12 | 0.00 | 1.00 | 0.03 | 0.00 | 1.00 |
| 6B | 22 | 212 | 125.59 | 0.13 | 0.00 | 1.00 | 0.03 | 0.00 | 1.00 |
| 6C | 23 | 104 | 18.59 | 0.21 | 0.00 | 1.00 | 0.03 | 0.00 | 1.00 |
| 6CII | 23 | 101 | 17.25 | 0.37 | 0.00 | 1.00 | 0.03 | 0.00 | 1.00 |
| 6D | 24 | 91 | 74.12 | 0.18 | 0.00 | 1.00 | 0.03 | 0.00 | 1.00 |
| 7A | 25 | 343 | 99.45 | 0.11 | 0.00 | 1.00 | 0.03 | 0.00 | 1.00 |
| 7B | 26 | 90 | 85.48 | 0.14 | 0.00 | 1.00 | 0.02 | 0.00 | 1.00 |
| 7C | 27 | 133 | 78.31 | 0.17 | 0.00 | 1.00 | 0.04 | 0.00 | 1.00 |
| 7D | 28 | 255 | 55.41 | 0.12 | 0.00 | 1.00 | 0.02 | 0.00 | 1.00 |
|  |  | *4841* | *1695.46* | 0.26 | 0.02 | 0.99 | 0.04 | 0.00 | 0.95 |

**Supplementary Table S5.** Selection efficiency for genetic gain of the 5%, and 10% top genotypes (corresponding to 15, and 30) according to three GS models fitted with T2/2013 data for training and used to make predictions in T2/2014. All numbers are based on GEBV estimated in T2/2014. AWT: average weight (g/fruit), EMY: early marketable yield (g/plant), SSC: soluble solids content (%), TC: proportion of total culls (%), TMY: total marketable yield (g/plant).

| Method |  | Trait |  | Selection Efficiency (%) | |  | Selected Genotypes | |
| --- | --- | --- | --- | --- | --- | --- | --- | --- |
|  |  |  |  | 5% | 10% |  | 5% | 10% |
| PBLUP |  | AWT |  | 66.4 | 59.7 |  | 6/15 | 11/30 |
|  |  | SSC |  | 53.3 | 57.8 |  | 3/15 | 11/30 |
|  |  | EMY |  | 36.3 | 23.4 |  | 3/15 | 5/30 |
|  |  | TMY |  | 24.2 | 14.2 |  | 2/15 | 5/30 |
|  |  | TC |  | 40.6 | 25.0 |  | 2/15 | 6/30 |
| GBLUP |  | AWT |  | 74.4 | 71.1 |  | 8/15 | 14/30 |
|  |  | SSC |  | 51.7 | 52.6 |  | 3/15 | 9/30 |
|  |  | EMY |  | 30.8 | 27.5 |  | 2/15 | 4/30 |
|  |  | TMY |  | 28.1 | 38.8 |  | 2/15 | 7/30 |
|  |  | TC |  | 48.4 | 51.5 |  | 4/15 | 8/30 |
| Bayes B |  | AWT |  | 70.4 | 73.7 |  | 7/15 | 15/30 |
|  |  | SSC |  | 55.8 | 52.9 |  | 4/15 | 9/30 |
|  |  | EMY |  | 31.5 | 33.5 |  | 2/15 | 5/30 |
|  |  | TMY |  | 46.5 | 45.8 |  | 6/15 | 11/30 |
|  |  | TC |  | 49.9 | 55.0 |  | 3/15 | 10/30 |

**SUPPLEMENTARY FIGURES**


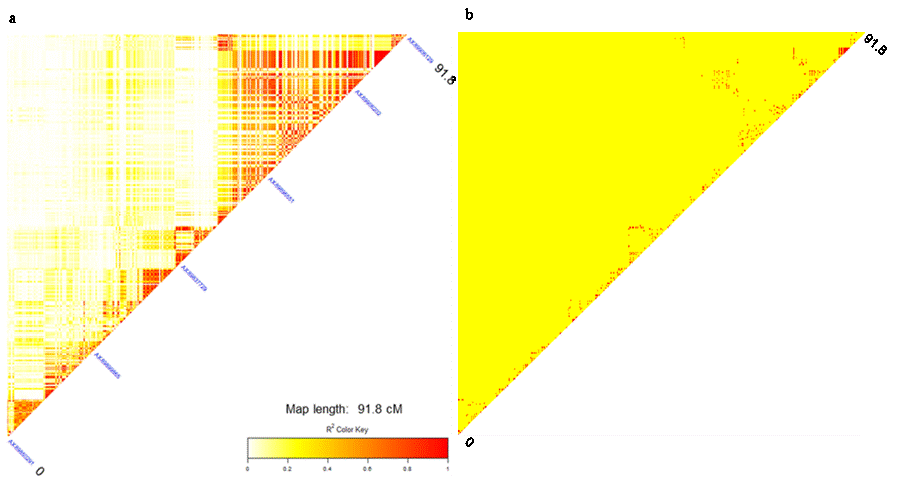


**Supplementary Figure S1.** Linkage disequilibrium (LD)
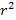
 and LD corrected for genetic relatedness
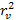
 for linkage group (LG) 6A in the T2/2013 population. (**a**) Heat map (upper diagonal matrix) of pairwise regular LD (
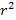
) for LG 6A. Marker names are anchored on the diagonal line: AX-89850291 is the 1st SNP marker at 0 cM. Next 100th, 200th, 300th, 400th and last marker AX-89906129 at 91.8 cM are also displayed at the diagonal line. (**b**) Heat map (upper diagonal matrix) of pairwise LD corrected for genetic relatedness
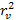
 for LG 6A.


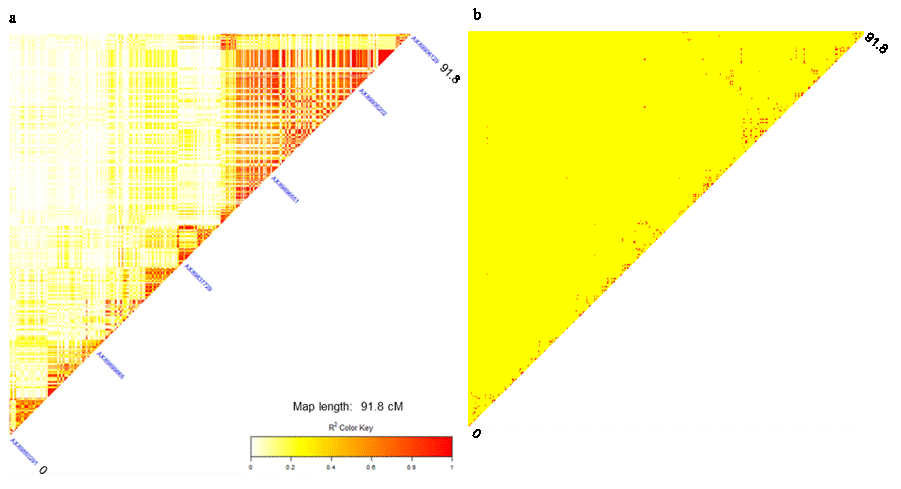


**Supplementary Figure S2.** Linkage disequilibrium (LD)
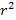
 and LD corrected for genetic relatedness
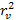
 for linkage group (LG) 6A in the T2/2014 population. (**a**) Heat map (upper diagonal matrix) of pairwise regular LD (
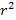
) in LG 6A. Marker names are anchored on the diagonal line: AX-89850291 is the1st SNP marker at 0 cM. Next 100th, 200th, 300th, 400th and last marker AX-89906129 at 91.8 cM are also displayed at the diagonal line. (**b**) Heat map (upper diagonal matrix) of pairwise LD corrected for genetic relatedness
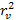
 in LG 6A.
